# Use of psychoactive drugs predicts functional disability among older adults

**DOI:** 10.11606/S1518-8787.2019053000675

**Published:** 2019-01-18

**Authors:** Denise Mourão Falci, Juliana Vaz de Melo Mambrini, Érico Castro-Costa, Josélia Oliveira Araújo Firmo, Maria Fernanda Lima-Costa, Antônio Ignácio de Loyola

**Affiliations:** I Instituto René Rachou. Fundação Oswaldo Cruz. Programa de Pós-Graduação em Saúde Coletiva. Belo Horizonte, MG, Brasil; II Instituto René Rachou. Fundação Oswaldo Cruz. Núcleo de Estudos em Envelhecimento e Saúde Pública. Belo Horizonte, MG, Brasil; III Universidade Federal de Minas Gerais. Escola de Enfermagem. Departamento de Enfermagem Aplicada. Belo Horizonte, MG, Brasil

**Keywords:** Aging, Aged, Psychoactive Drugs, Disabled Persons, Cohort Studies, Activities of Daily Living, Envelhecimento, Idoso, Psicofármacos, Pessoas com Deficiência, Estudos de Coortes, Atividades de Vida Diária

## Abstract

**OBJECTIVE:**

Investigate whether the use of psychoactive drugs would be a predictor of incidence of functional disability among seniors living in community.

**METHODS:**

It is a population-based longitudinal study, developed between January 1, 1997 and December 31, 2011, with older adults living in community. The association between the use of psychoactive drugs and the development of functional disability for instrumental (IADLs) and basic (BADLs) activities of daily living was tested using the extended Cox proportional hazards model, which considers the measure of exposure of interest throughout the follow-up period. The analyses were stratified by sex and adjusted by sociodemographic characteristics, health behavior and health conditions.

**RESULTS:**

After multivariate adjustment, the use of two or more psychoactive drugs in the female stratum was associated with disability for both IADLs (HR = 1.58; 95%CI 1.17–2.13) and BADLs (HR = 1.43; 95%CI 1.05–1.94), the use of benzodiazepines was associated with disability for IADLs (HR = 1.32; 95%CI 1.07–1.62), and the use of antidepressants was associated with disability for both IADLs (HR = 1.51; 95%CI 1.16–1.98) and BADLs (HR = 1.44; 95%CI 1.10–1.90). In the male stratum, the use of antipsychotics was associated with disability for IADLs (HR = 3.14; 95%CI 1.49–6.59).

**CONCLUSIONS:**

The study showed a prospective association between the use of psychoactive drugs and functional disability. These results indicate the need to carefully assess the prescription of psychoactive drugs for older adults and monitor their usage in order to detect damages to the health of users.

## RESUMO

**OBJETIVO:**

Investigar se o uso de psicofármacos seria um preditor da incidência de incapacidade funcional entre idosos residentes em comunidade.

**MÉTODOS:**

Trata-se de um estudo longitudinal de base populacional, desenvolvido entre primeiro de janeiro de 1997 e 31 de dezembro de 2011, junto a idosos residentes em comunidade. A associação entre o uso de psicofármacos e o desenvolvimento de incapacidade funcional para atividades instrumentais (AIVD) e básicas (ABVD) de vida diária foi testada por meio do modelo de riscos proporcionais de Cox estendido, que considera a medida da exposição de interesse ao longo de todo o tempo de seguimento. As análises foram estratificadas por sexo e ajustadas por características sociodemográficas, comportamento em saúde e condições de saúde.

**RESULTADOS:**

Após ajuste multivariado, no estrato feminino o uso de dois ou mais psicofármacos foi associado à incapacidade tanto para AIVD (HR = 1,58; IC95% 1,17–2,13) quanto para ABVD (HR = 1,43; IC95% 1,05–1,94), o uso de benzodiazepínicos se manteve associado à incapacidade para AIVD (HR = 1,32; IC95% 1,07–1,62) e o uso de antidepressivos se manteve associado à incapacidade, tanto para AIVD (HR = 1,51; IC95% 1,16–1,98) quanto para ABVD (HR = 1,44; IC95% 1,10–1,90). No estrato masculino, o uso de antipsicóticos foi associado à incapacidade para AIVD (HR = 3,14; IC95% 1,49–6,59).

**CONCLUSÕES:**

O estudo evidenciou uma associação prospectiva entre o uso de psicofármacos e a incapacidade funcional. Esses resultados indicam a necessidade de avaliar cuidadosamente a prescrição de psicofármacos para idosos e monitorar o seu uso, buscando detectar prejuízos à saúde dos seus usuários.

## INTRODUCTION

Low-income countries, such as Brazil, have aged fast. It is estimated that 80% of the population of these countries will be composed of older adults by the half of this century, posing a challenge to ensure the gains in longevity mean additional years with health and quality of life^1^. For the older adult, welfare and quality of life are more strongly linked to the preservation of functional capacity than to the absence of diseases^2^.

Functional disability can be understood as the loss of ability to perform everyday tasks necessary for an independent and autonomous life. These activities are grouped into two sets, one concerning activities related to self-care and survival (basic activities) and other regarding activities of life in society (instrumental activities)^3^. A wide range of factors contribute to the development of functional disability, from sociodemographic characteristics (especially age) to health conditions (chronic diseases commonly present in old age)^4^.

Older adults use medicines more frequently and intensely than younger adults^5^ and psychoactive drugs are among the most used ones by this populational segment^6^. Psychoactive drugs act directly on the central nervous system and are used in the treatment of mood and behavior disorders^7^. The possible involvement of psychoactive drugs in the genesis of functional disability among older adults has been investigated in several international studies^8^ ^-^ ^12^, but the literature does not register Brazilian studies with such purpose.

This longitudinal study aimed to investigate whether the use of psychoactive drugs would be a predictor of the incidence of functional disability among seniors living in community.

## METHODS

### Study Area and Population

This research integrates the Bambuí Project, a population-based longitudinal study on healthy aging, developed at the headquarters of the municipality of the same name, located in the southwest region of the state of Minas Gerais, Brazil.

At the time of the Bambuí Cohort Study on Aging (1997), the population of the municipality totaled 21,187 inhabitants, of which 70.0% (approximately 15,000 inhabitants) lived at the headquarters of the municipality. All residents in the town aged 60 years or older on January 1, 1997 were invited to participate in the cohort baseline, totalizing 1,742 older adults. Of these, 1,606 (92.2%) people were interviewed, representing the target population of the study. More details can be found in other publication^13^.

**Figure 1:**
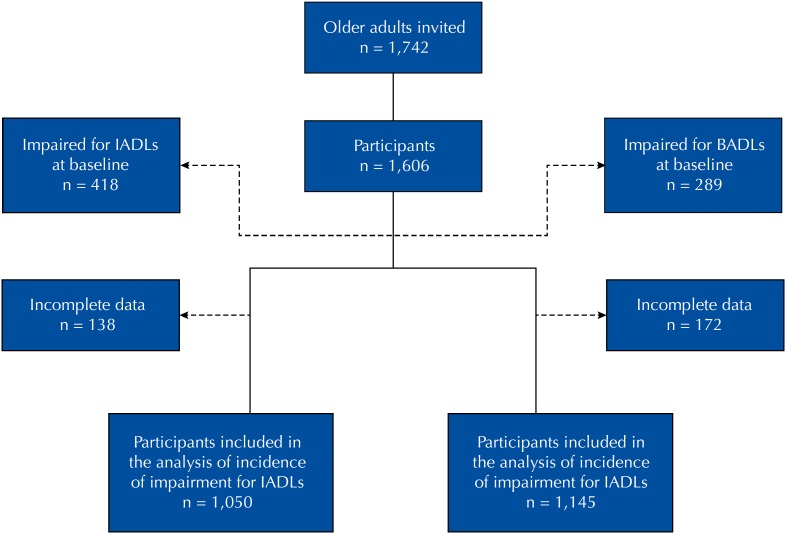
Flowchart of the study population, Bambuí, state of Minas Gerais, Brazil, 1997–2011. IADLs: instrumental activities of daily living; BADLs: basic activities of daily living

Of the older adults participating in the cohort study, all those able to perform instrumental activities of daily living (IADLs) or basic activities of daily living (BADLs) at baseline were considered eligible for this study. Thus, 1,050 and 1,145 participants were included in the longitudinal analysis of incidence of functional disability for IADLs and BADLs, respectively (Figure 1). Among those free from disability for IADLs, 639 (60.9%) composed the female stratum and 411 (39.1%), the male stratum; among those free from disability for BADLs, these numbers were 702 (61.3%) and 443 (38.7%), respectively.

### Study Variables and Data Collection

Data were collected using a questionnaire, applied in interviews conducted in the households. Each interview lasted approximately 40 minutes and could be completed in one or two visits as needed by the respondent.

### Variable – Outcome

The domains of functional disability were investigated separately. The incidence of functional disability for both domains was verified every year (between 1998 and 2011) based on self-report of the difficulty level (none; some; very; cannot perform) for instrumental or basic activities. Disability for IADLs and BADLs were measured based on the scales of Lawton and Brody^14^ and Katz^15^, respectively. IADLs included in the study were: to perform housework (sweep the house, remove dust, use vacuum cleaner); cook their own meals; manage their own money (to control their expenses or pay off their bills); and go out shopping. The BADLs covered were: walk from a room to another on the same floor; get up from a chair without arm; rise from bed; eat with a fork, cut food, drink from a cup; dress up, as well as to put on shoes, close a zipper, close and open buttons; go to the bathroom in time; and take a shower.

Participants who had a lot of difficulty or who could not perform at least one of the activities investigated for each domain were considered disabled. The incidence of disability for IADLs and BADLs were investigated in separate databases; in both of them, the variables were treated dichotomously (independent – code 0; dependent – code 1).

### Population of Interest

The exposure of interest was the use of psychoactive drugs, whose measurement was based on the following question: “In the last 90 days, did you take any medicine? It does not matter whether it was prescribed by a physician or the reason why you are taking the medicine.” To minimize the loss of information, the conference of prescriptions and packaging was held.

The medicines mentioned were identified and unfolded in their active principles. From their chemical formulation, they were classified based on the Anatomical Therapeutic Chemical Index (ATC/DDD), developed by the World Health Organization Collaborating Center for Drug Statistics Methodology^a^. We classified as psychoactive the drugs that fit in the following ATC codes: N05A (antipsychotics), N05B (anxiolytics), N05C (hypnotics and sedatives), N06A (antidepressants); N06C (psycholeptics and psychoanaleptics in combination), and N06D (antidementia drugs). Clonazepam, classified by ATC as antiepileptic (N03A), was also considered psychoactive (benzodiazepine), for being routinely prescribed as anxiolytic for anxiety-related sleep disorders. Based on this criterion, the exposure variables of interest were defined, which were the number of psychoactive drugs used (0; 1; ≥ 2) and descriptive variables of the use of specific therapeutic classes (benzodiazepines, antidepressants, antipsychotics), all dichotomized.

### Adjustment variables

The variables included in the study for the adjustment purpose covered sociodemographic characteristics, as well as health behavior and health conditions. These variables were selected because they were consistently associated with the event and to the exposure of interest. The sociodemographic characteristics included age (60–69; 70–79; ≥ 80), schooling in complete years of school attendance (0–3; ≥ 4) and marital status (married; widower, single or divorced). Descriptive variables of health behaviors and health conditions were smoking (never smoked; former smoker; smoker); obesity (dichotomous); self-rated health (very good or good; reasonable; bad); number of chronic conditions (0; 1; ≥ 2); insomnia complaints, presence of depressive symptoms and cognitive dysfunction (the last three dichotomized). Individuals with body mass index (BMI) ≥ 30 kg/m^2^ were classified as obese^16^. Chronic diseases included Chagas disease, diabetes, hypertension, arthritis or rheumatism, angina, and cerebrovascular accident (CVA). The presence of depressive symptoms was evaluated using the General Health Questionnaire^17^ and a score of ≥ 5 as a positive cutoff point. Cognitive dysfunction was researched using the mini-mental state examination, being positive the participant with a score lower than 22^18^.

### Data Analysis

The measures of exposure of interest (use of psychoactive drugs) were held at baseline (1997) and in all follow-ups (1998 to 2011). The adjustment variables were measured only at the baseline. Initially, the characteristics of older adults with disability for IADLs or for BADLs were compared with the independent ones (reference category), using the Pearson's Chi-squared test. Incidence density rate (per 1,000 persons-year) were calculated for disability on IADLs or BADLs in the total population and in the female and male population strata. Kaplan-Meier curves were constructed to describe the incidence of disability for IADLs and BADLs over time, according to sex. Hypotheses of association between the use of psychoactive drugs and functional disability for each one of the domains (IADLs and BADLs) were tested using the univariate and multivariate analyses. We conducted separate analyses for each exposure variable of interest (number of psychoactive drugs, benzodiazepines, antidepressants and antipsychotics) using the extended Cox proportional hazards model, stratified by sex. The Cox model estimates the hazard ratio (HR) and respective confidence intervals at 95% (95%CI), and the premise of risk proportionality over time was verified. The extended model considers the time-dependent exposure measure, i.e. any variations in exposure over the follow-up period are contemplated, not just baseline information, which results in more precise association measures. The criterion of statistical significance was 5%. Deaths and losses were considered censoring, and the failure (event) was defined after the occurrence of the outcome investigated, regardless of any future functional recovery of the participant. The analyses were performed using the Stata^®^ software, version 14.0 (Stata Corp., College Station, USA).

### Ethical Considerations

The Bambuí Project was approved by the Ethics Committee of the Oswaldo Cruz Foundation, and all participants signed a free and informed consent form.

## RESULTS

For this research, two study populations were established: older adults free from functional disability for IADLs (n = 1,050) and older adults free from functional disability for BADLs (n = 1,145). Both populations presented a similar distribution of adjustment variables and of the use of psychoactive drugs by sex at the study baseline (Table 1). Men and women were similar regarding age and schooling (predominance of the age group between 60-69 years and schooling less than four years), but the proportion of unmarried older women was significantly higher. Regarding health behaviors and health conditions, differences by sex were observed for obesity, worse self-rated health, number of chronic diseases, insomnia complaints and depressive symptoms (more frequent among women), as well as for smoking and cognitive dysfunction (more frequent among men). In both populations, the prevalence of psychoactive drugs was significantly higher (p < 0.05) among women, who took benzodiazepines, antidepressants and antipsychotics more frequently, in that order.

**Table 1:** Distribution of adjustment variables and use of psychoactive drugs at the baseline (1997) stratified by sex. Bambuí, state of Minas Gerais, Brazil.

Variable		Impairment for IADLs (n = 1,050)			Impairment for BADLs (n = 1,145)		
		Male (n = 411)	Female (n = 639)	p*	Male (n = 443)	Female (n = 702)	p*
Age (years)				0.439			0.488
	60–69	65.9	63.5		64.6	61.3	
	70–79	27.3	30.7		28.2	31.5	
	≥ 80	6.8	5.8		7.2	7.3	
Schooling (years)				0.248			0.064
	0–3	58.9	62.4		57,3	62.8	
	≥ 4	41.1	37.6		42.7	37.2	
Marital status				< 0.001			< 0.001
	Married	76.6	34.7		76.1	34.1	
	Widowed	11.4	48.8		11.7	48.9	
	Single or divorced	11.9	16.4		12.2	17.1	
Obesity				< 0.001			< 0.001
	No	94.4	84.0		94.6	85.9	
	Yes	5.6	16.0		5.4	14.1	
Smoking				< 0.001			< 0.001
	Never	31.9	79.5		29.8	78.6	
	Former smoker	41.6	10.2		42.4	10.8	
	Current smoker	26.5	10.3		27.8	10.5	
Self-assessment of health				0.096			0.039
	Very good or good	44.0	37.6		43.6	36.9	
	Reasonable	44.3	48.2		44.5	47.2	
	Bad	11.7	14.2		12.0	16.0	
No. of chronic diseases				< 0.001			< 0.001
	0	18.7	12.4		18.1	12.0	
	1	43.6	36.6		43.3	36.0	
	≥ 2	37.7	51.0		38.6	52.0	
Insomnia complaints				< 0.001			< 0.001
	No	75.4	57.9		75.4	57.4	
	Yes	24.6	42.1		24.6	42.6	
Depressive symptoms				< 0.001			< 0.001
	No	76.6	63.5		76.5	62.0	
	Yes	23.4	36.5		23.5	38.0	
Cognitive dysfunction				< 0.001			0.001
	No	80.3	88.3		79.5	86.6	
	Yes	19.7	11.7		20.5	13.4	
No. of psychoactive drugs				< 0.001			< 0.001
	0	84.9	68.9		84.4	66.5	
	1	11.7	22.7		11.5	24.4	
	≥ 2	3.4	8.5		4.1	9.1	
Use of benzodiazepines				< 0.001			< 0.001
	No	87.6	75.4		87.4	73.2	
	Yes	12.4	24.6		12.6	26.8	
Use of antidepressants				< 0.001			< 0.001
	No	97.1	89.7		96.4	89.5	
	Yes	2.9	10.3		3.6	10.5	
Use of antipsychotic drugs				0.012			0.007
	No	98.5	95.8		98,4	95.4	
	Yes	1.5	4.2		1.6	4.6	

IADLs: instrumental activities of daily living; BADLs: basic activities of daily living

* Pearson's Chi-squared test, significative when p < 0.05.

**Figure 2:**
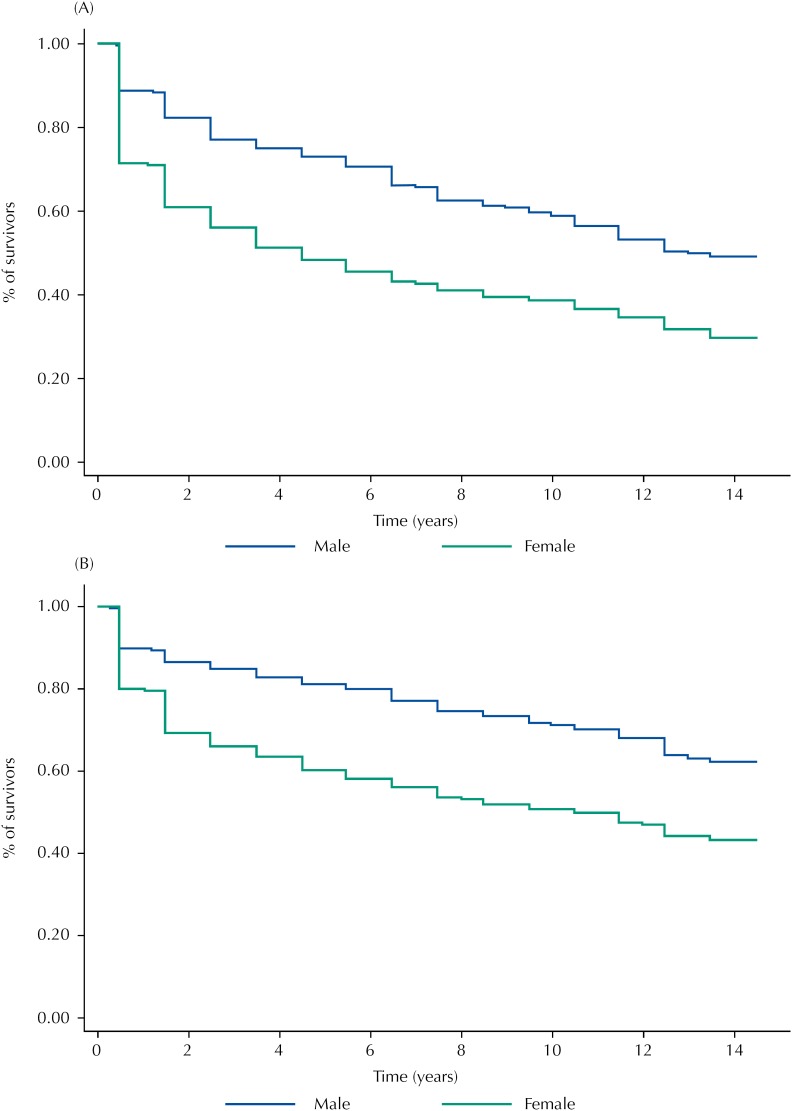
Kaplan-Meier curve of the incidence of impairment for IADLs (A) and BADLs (B), by sex. Bambuí, state of Minas Gerais, Brazil, 1997–2011. IADLs: instrumental activities of daily living; BADLs: basic activities of daily living

In this study, individuals free from disability for IADLs contributed to 7,212 person-year of observation, while among those free from disability for BADLs, the total was 8,944 person-year. Over the follow-up period, 609 (58.0%) participants developed functional disability for some IADLs and 509 (44.5%) for some BADLs, resulting in Incidence density rate of 84.4/1,000 person-year and 56.9/1,000 person-year, respectively. Incidence density rates (per 1,000 person-year) for both IADLs and BADLs were higher among women (109.3 for IADLs and 72.7 for BADLs) than among men (54.9 for IADLs and 36.0 for BADLs). Figure 2 depicts the Kaplan-Meier survival curve for both IADLs (Figure 2, A) and BADLs (Figure 2, B). Over the period, the proportion of older adults free from both disabilities was consistently higher among men than among women. Regarding IADLs, 50% of the women were unabled within four and a half years of follow-up, whereas among men this occurred only after 13 years of follow-up. As to the disability for BADLs, half of the women were unabled after 10.5 years of follow-up. At the end of the study, just over 60% of men remained free from disability for BADLs.

Tables 2 and 3 shows the results of the univariate and multivariate analyses of the association between the use of psychoactive drugs and functional disability for both domains, stratified by sex. Regarding IADLs, only the use of antipsychotics (HR = 3.14, 95%CI 1.49–6.59) was an independent predictor of disability among men. Among women, a positive and independent association was observed for the overall use of psychoactive drugs (HR = 1.34, 95%CI 1.08–1.67 for a psychoactive drug and HR = 1.58, 95%CI 1.17–2.13 for two or more psychoactive drugs), benzodiazepines (HR = 1.32, 95%CI 1.07–1.62) and antidepressants (HR = 1.51, 95%CI 1.16–1.98) (Table 2).

**Table 2:** Univariate and multivariate analyses of the association between the use of psychoactive drugs and functional impairment for IADLs, stratified by sex. Bambuí, state of Minas Gerais, Brazil, 1997–2011.

Use of psychoactive drug		Impairment for IADLs	
		HR (95%CI) crude	HR (95%CI) Adjusted^c^
Male			
No. of psychoactive drugs			
	1	1.64 (1.15–2.34)^a^	1.38 (0.96–2.00)
	≥ 2	1.83 (0.93–3.61)	1.40 (0.71–2.79)
	Benzodiazepine	1.44 (0.97–2.13)	1.17 (0.78–1.75)
	Antidepressant	1.35 (0.71–2.57)	1.23 (0.64–2.37)
	Antipsychotic	4.23 (2.07–8.62)^b^	3.14 (1.49–6.59)^a^
Female			
No. of psychoactive drugs			
	1	1.44 (1.16–1.79)^a^	1.34 (1.08–1.67)^a^
	≥ 2	1.63 (1.22–2.19)^a^	1.58 (1.17–2.13)^a^
	Benzodiazepine	1.41 (1.14–1.73)^a^	1.32 (1.07–1.62)^a^
	Antidepressant	1.50 (1.15–1.95)^a^	1.51 (1.16–1.98)^a^
	Antipsychotic	1.26 (0.78–2.06)	1.26 (0.77–2.06)

HR (95%): hazard ratio (95% confidence interval), estimated by extended Cox regression; IADLs: instrumental activities of daily living; BADLs: basic activities of daily living

In each stratum, separate models were tested for each of the exposures: number of psychoactive drugs, benzodiazepines, antidepressants and antipsychotics.

a p < 0.05

b p < 0.001

c Adjusted by age, schooling, marital status, obesity, smoking, self-assessment of health, number of chronic diseases, insomnia complaints, depressive symptoms and cognitive dysfunction.

**Table 3:** Univariate and multivariate analyses of the association between the use of psychoactive drugs and functional impairment for BADLs, stratified by sex. Bambuí, state of Minas Gerais, Brazil, 1997–2011.

Use of psychoactive drug		Impairment for BADLs	
		HR (95%CI) crude	HR (95%CI) adjusted^a^
Male			
No. of psychoactive drugs			
	1	1.19 (0.77–1.85)	1.00 (0.63–1.57)
	2+	1.15 (0.53–2.47)	0.85 (0.39–1.86)
	Benzodiazepine	1.45 (0.94–2.24)	1.09 (0.69–1.72)
	Antidepressant	1.01 (0.47–2.17)	0.92 (0.43–1.99)
	Antipsychotic	0.60 (0.15–2.41)	0.51 (0.13–2.10)
Female			
No. of psychoactive drugs			
	1	1.41 (1.12–1.78^b^)^b^	1.21 (0.96–1.54)
	2+	1.56 (1.15–2.10)^b^	1.43 (1.05–1.94)^b^
	Benzodiazepine	1.33 (1.07–1.65)^b^	1.17 (0.94–1.46)
	Antidepressant	1.49 (1.14–1.95)^b^	1.44 (1.10–1.90)^b^
	Antipsychotic	1.43 (0.90–2.27)	1.43 (0.90–2.28)

HR (95%CI) Hazard Ratio (95% confidence interval), estimated by extended Cox regression; IADLs: instrumental activities of daily living; BADLs: basic activities of daily living

In each stratum, separate models were tested for each of the exposures: number of psychoactive drugs, benzodiazepines, antidepressants and antipsychotics.

a Adjusted by: age, schooling, marital status, obesity, smoking, self-assessment of health, number of chronic diseases, insomnia complaints, depressive symptoms and cognitive dysfunction.

b p < 0.05

As for the domain of BADLs, the use of psychoactive drugs was not associated with disability among men. Among women, the use of two or more psychoactive drugs (HR = 1.43; 95%CI 1.05–1.94) and antidepressants (HR = 1.44; 95%CI 1.10–1.90) were independent predictors of disability for BADLs (Table 3).

## DISCUSSION

This study provides additional evidence that psychoactive drugs are associated with the development of functional disability among older adults, both for IADLs and BADLs, and that these associations vary according to the sex of the older adult and quantity and class of the psychoactive drugs used. In the female stratum, with the exception of antipsychotics, the other psychoactive drugs investigated were associated with the development of functional disability, for both IADLs (benzodiazepines and antidepressants) and BADLs (only antidepressants). In the same stratum, evidence of dose-response effect regarding the association between the number of psychoactive drugs used and impairment for IADLs was detected. Among men, the antipsychotics were the only psychoactive drugs to predict functional disability, restricted to IADLs.

Benzodiazepines are the psychotropic drugs that have been most investigated for their potential impairment in functional capacity among older adults, from a longitudinal perspective. Among French older people, chronic users of benzodiazepines have a higher risk to develop limitations in IADLs^10^; our results of the female stratum corroborate this French study. Associations between the use of benzodiazepines and the incidence of disability for BADLs were observed in other older populations^11^ ^,^ ^12^ ^,^ ^19^, even after adjusting for broad set of covariates (sociodemographic, health behavior and health conditions). In our study, although the risk of impairment for IADLs was higher among older adults who took benzodiazepines, the association was not significant.

The likely explanation for the relationship between the use of benzodiazepines and the onset of functional disability for IADLs derives from their pharmacological action and adverse effects. Benzodiazepines stand out for their sedative and hypnotic action. The cumulative effect of the sedative action affects the physical movements and motor coordination, damaging the psychomotor performance. Among older adults, the benzodiazepines are related to the cognitive impairment, as well as to the occurrence of delirium, falls and fractures^20^.

Among women (but not among men), the use of antidepressants has contributed to the development of disability for both types of activities of daily living, the instrumental and the basic ones, in similar intensity (increased the risk for IADLs in 51% and for BADLs in 44%). Our findings contradict that observed in a study on Chinese institutionalized older adults with depressive disorders, in which the use of antidepressants was a protection factor against the decline of the functional capacity^21^. However, our results corroborate, in part, the findings of another recent study, developed with American adults (50 years or older), in which the use of antidepressants was a predictor of functional impairment for BADLs, although it was not a predictor for IADLs^8^.

Even though their effectiveness in the treatment of depressive disorders is recognized, there is evidence that adverse effects of antidepressants impair the functionality among older adults^22^. Many tricyclic antidepressants (amitriptyline, clomipramine, paroxetine, among others) have anticholinergic properties, which have been linked to the harm of cognitive and motor functions^23^. In addition, the prolonged use of some antidepressants represents greater risk of falling and hip fractures^24^, events that can lead to disability, especially among women as a result of the loss of bone mass due to hormonal actions^25^. Cognitive ability is more heavily involved in the IADLs. On the other hand, the completion of BADLs is more mechanical and depends on a good physical performance, such as muscle strength and motor coordination, which can be compromised by the use of these medicines. Thus, the deleterious effects of antidepressants on the cognitive and physical functions would harm the functional capacity, both for IADLs and BADLs.

Among men, the antipsychotics were the only class of psychoactive drugs to predict functional disability for IADLs. The risk of impairment for IADLs among older adults who reported use of antipsychotics was the triple of that measured among those who did not take this medicine. This association was also detected between Italian institutionalized seniors (residents in nursing homes), followed during 12 months^9^. The direct comparison of the results is hindered by the differences between study populations: the participants in the study mentioned above were older and had a higher frequency of comorbidities than the participants in our study. Antipsychotics are drugs used in the management of behavioral and psychological symptoms of psychotic disorders and dementia, such as agitation and aggressiveness^26^. Symptoms of psychotic disorders are more exacerbated among men^27^. Just as in the case of other psychoactive drugs already discussed, the antipsychotics are considered inappropriate for older adults^20^, exhibiting side effects such as sedation, extrapyramidal effects and dizziness, which increases the risk of a fall and worsens the motor and cognitive functions^28^. In the same way as the other psychoactive drugs here investigated, the relationship between the use of antipsychotics and impairment can be related to the adverse effects. It is necessary to pay attention to the results that associate antipsychotics with disability due to the frequency with which this medication is taken among older adults^29^ and due to their off-label use in addressing health problems other than psychotic disorders^30^.

The use of more than one psychoactive drug was associated with disability for both BADLs and IADLs, although this association has been restricted to the female stratum. This association is consistent with the fact that women took higher amounts of psychoactive drugs and that, in the female stratum, more than one class of psychoactive drug was associated with functional disability.

To prevent confusion, our analysis considered a wide range of variables, including sociodemographic characteristics, use of healthcare services and health conditions. Among the latter ones, disabling diseases (such as diabetes, Chagas disease and arthritis) and mental disorders whose pharmacological approach is made with psychoactive drugs were included, as in the case of depressive symptoms and sleep disorders. However, we did not include measures of psychotic disorders, which does not rule out the confusion by indication in the association between the use of antipsychotics and disability. It is possible that the association observed between the use of antipsychotics and disability for IADLs is partially explained by behavioral symptoms typical of psychotic disorders such as aggressiveness and social isolation. The IADLs are typical of life in society, and they require social interaction. In the same line of thought, the non-inclusion of the variable fall (associated with the psychiatric drugs investigated) hinders a clearer understanding as to the possible contribution of this event for the development of functional disability in this study population. In any case, the association detected indicates the possibility of the drug being at the origin of the causal chain that would culminate in the appearance of the disability.

On the other hand, the strength of this study derives from the methodological approach adopted, such as the use of time-dependent exposure variable. This allowed us to capture the dynamics of the exposure over the 14 years of follow-up, which included the beginning of the use of the medicine at a different time from the baseline, or even its discontinuity, resulting in more accurate measures of association. In addition, the use of psychoactive drugs was based on self-report, which provides a picture of the use of drugs as close to the reality, besides verifying the medical prescriptions and the packaging of medicines, in order to minimize memory problems. The self-report provides a measure closer to the effective use of medicine than the prescription and dispensing records, because they do not ensure that the medicine has been effectively used. Finally, it is worth referring to the innovative character of this study, as it is the first Brazilian study investigating the association between the use of psychoactive drugs and the functional disability under a longitudinal perspective.

In short, this population-based longitudinal study was the first, in Brazil, to highlight the contribution of the use of psychoactive drugs (in general or in specific classes) to the incidence of functional disability among older adults. To prevent the functional disability, or at least slow down its onset, it is essential to ensure that the gains in life expectancy result in more years spent with quality. Because the use of psychoactive drugs constitute a potentially modifiable risk factor, health professionals should carefully assess the relevance of their prescription. In this sense, to seek alternatives to pharmacological therapy may be a viable strategy to prevent disability and maintain the quality of life of the older adults. Considering the results of this study, which confirm findings in other older populations, and the inevitability of the use of psychoactive drugs, patients should be monitored and assessed routinely by their clinicians, so that the benefits of prescription are not outweighed by the risks involved in the use of these medicines.

## Footnotes

a World Health Organization. Anatomical therapeutical chemical (ATC) classification index with defined daily doses (DDDs). Geneva: WHO [cited 2016 Nov 10]. Available from: http://www.whocc.no/atc_ddd_index/
